# The associations between body dissatisfaction, exercise intensity, sleep quality, and depression in university students in southern China

**DOI:** 10.3389/fpsyt.2023.1118855

**Published:** 2023-03-20

**Authors:** Ming Hao, Xuesheng Liu, Ying Wang, Qingfeng Wu, Wenjing Yan, Yanbin Hao

**Affiliations:** ^1^School of Public Health and Health Management, Gannan Medical University, Ganzhou, China; ^2^Institute for the Prevention and Control of Infectious and Communicable Diseases, Liaoning Province Center for Disease Control and Prevention, Shenyang, China; ^3^Key Laboratory of Prevention and Treatment of Cardiovascular and Cerebrovascular Diseases, Ministry of Education, Gannan Medical University, Ganzhou, China

**Keywords:** depression, body dissatisfaction, exercise intensity, sleep quality, young people, southern China

## Abstract

**Background:**

In recent years, depression in early adulthood has become an urgent global public health concern. The university years mark a transitional period from adolescence to adulthood. Young people are required to face academic and life pressures independently, which increases the risk of mental health problems in university.

**Purpose:**

The main goal of the current study was to explore the sex differences in depression, body dissatisfaction, sleep quality, and exercise intensity among university students in southern China and to analyze the factors affecting the level of depression among university students.

**Methods:**

In total, 1,258 university students aged 18–23 years were recruited for this study. All participants completed anthropometric measurements, the Self-rating Depression Scale, Physical Activity Rating Scale, and Pittsburgh Sleep Quality Index. Body dissatisfaction levels were measured using sex-appropriate silhouettes.

**Results:**

Compared with young women, young men had higher exercise intensity and sleep quality, whereas young women’s body dissatisfaction and depression levels were significantly higher than those of young men. Sleep quality score (β = 0.34, *p* < 0.01), sex (β = 0.15, *p* < 0.01), physical activity score (β = −0.14, *p* < 0.01), and body dissatisfaction (β = 0.14, *p* < 0.01) were significant predictive factors of the Self-rating Depression Scale score.

**Conclusion:**

Low levels of physical dissatisfaction have a positive effect on depression, and high levels of physical activity and quality sleep can also improve depressive symptoms. At the same time, increasing body satisfaction has the effect of increasing physical activity and improving sleep quality. Therefore, there is great potential to prevent and ameliorate depression by reducing body dissatisfaction.

## 1. Introduction

As of 2018, the number of people with depression worldwide had reached 322 million (1). By 2030, depression will become one of the world’s most burdensome diseases (2). In China, 130 million adults suffer from mental illness each year on average (1). Depression is one of the most prevalent mental health disorders and can cause anxiety, sleep disturbances, and other adverse consequences. People with major depression sometimes show suicidal behavior (3).

Sleep disturbance is considered one of the most common symptoms of adolescent depression (4). Recent studies have found that sleep disturbance is also an important factor leading to the occurrence and existence of depression (5, 6). At present, the main methods for improving sleep are drugs and behavioral therapy. Because of the risk of dependence and tolerability, more patients are willing to choose non-drug therapy (7). Results of a meta-analysis of 49 (*n* = 5,908 subjects) non-pharmacological sleep intervention trials showed that treatment of sleep problems significantly reduced depressive symptoms (8). However, methods of treating insomnia, such as cognitive behavioral therapy, cannot be widely used to prevent and improve insomnia because they require the assistance of trained practitioners (9). Therefore, it is important to explore how to further improve sleep quality.

In addition, some studies have shown that exercise can positively affect mental health by reducing stress hormone levels (10). However, insufficient physical activity among adults is a serious problem. Based on a pooled analysis of 1.9 million respondents worldwide, 25% of adults fail to achieve adequate levels of physical activity (11). Studies have shown that only 10% of Chinese adults regularly perform physical exercise (12). Low physical activity level may be an important reason for the high incidence of psychological problems among adults (13–15).

Body image is defined as an individual’s perception of one’s own appearance and is mapped in the brain (16). It is thought to be linked to self-knowledge, self-attitudes, beliefs, thoughts, emotions, and behaviors (17). Body image can be explained as one’s mental image of the body and feeling of body shape (18). When there is a difference between the actual body and idealized body, people are dissatisfied with their body shape. Body dissatisfaction is widespread among children and adults; however, the widespread problem of body dissatisfaction has not received enough attention (19). Few studies have examined the relationship between body dissatisfaction and depression among university students.

The university years mark a transitional period from adolescence to adulthood. Young people are required to face academic and life pressures independently, which increases the risk of mental health problems in university (20). University students are more likely to develop mental disorders than other age groups (21). In a meta-analysis of 24 papers published between 1990 and 2010, which surveyed more than 10 countries and regions including the United States, Canada, China, and South Korea, the authors speculated that the average prevalence of mental disorders among university students worldwide reached 30.6% (22). In recent years, there have been many reports in different countries regarding the high incidence of depression among university students. A 3-year survey in a university in the United States showed that 29.6% of the university students suffered from mild depression and 6.6% suffered from major depression (23). It has been reported that British university students suffer from a high rate of depression (24). In Northern Ireland, the reported incidence of major depressive disorder was 24% and that of general anxiety disorder was 23%; 31% of the participants had thoughts of suicide, and 1 in 5 had made suicide plans (3). It is estimated that the prevalence of depression among university students in China is 23.8% (25). Therefore, improving depression status in early adulthood has become an urgent global public health issue. The main goal of the current study is to investigate sex differences in depression, body dissatisfaction, sleep, and exercise among university students in southern China and to analyze the body dissatisfaction affecting the level of depression among university students.

## 2. Materials and methods

### 2.1. Participants

We selected a comprehensive university in Ganzhou, Jiangxi Province, China. Participant recruitment information was disseminated on campus through posters put up in front of the dormitory building and leaflets distributed in study rooms. We recruited a total of 1,258 university students aged 18–23 years (men: 630; women: 628), who voluntarily signed and submitted their consent to be research subjects. The investigation period was from September to December, 2021.

### 2.2. Measurements

A portable stadiometer (Seca 213) with an accuracy of 0.1 cm was used to measure height. Weight (0.1 kg precision), muscle mass (0.1 kg precision), and fat percentage were measured using a body composition meter (BC 601, Tanita). Body mass index (BMI) was calculated as weight/height^2^ (kg/m^2^).

### 2.3. Body image

Body dissatisfaction levels were measured using a Stunkard visual graph table (26). The scale is based on a 9-point scale, with 1–9 representing body types for each sex and values ranging from the lowest (1) indicating a “very thin” body type to the highest (9) indicating a “very fat” body type. University students were asked to choose between what they considered their “current body type” and their “ideal body type.” The body dissatisfaction level represents the difference between current and ideal bodies and does not pertain to the preference for fat or thin.

### 2.4. Self-rating depression scale

The Self-rating Depression Scale (SDS) was used to measure the depression levels of the university students (27). It contains 20 items and is divided into four grades. The standard score is obtained by multiplying the sum of each individual score by 1.25; the higher the standard score, the more severe the depressive symptoms.

### 2.5. Physical activity rating scale

We used the Physical Activity Rating Scale (PARS-3) to evaluate the athletic level of the university students (28). The scale examines exercise with regard to three items: intensity, time, and frequency of exercise, and points ranging from 1 to 5 are assigned for each item. The formula for calculating the total exercise score is intensity × (time − 1) × frequency, and the result ranges from 0 to 100 points; the higher the score, the higher is the exercise intensity.

### 2.6. Sleep status scale

We measured sleep quality using the Pittsburgh Sleep Quality Index (PSQI), a 19-item scale. This scale can be used to assess sleep quality in the past month and contains seven component scores: sleep latency, sleep duration, sleep disturbance, daytime dysfunction, habitual sleep efficiency, sleep medication, and overall sleep quality (29). The PSQI score is a combination of these seven components, with higher scores indicating poorer sleep quality.

### 2.7. Sociodemographic characteristics

Data on the age, sex, and monthly cost of living of the university students were collected through a questionnaire.

### 2.8. Statistical methods

We used the independent sample *t*-test to verify BMI, body fat percentage, muscle mass, depression score, sleep quality score, exercise intensity score, and sex differences in the level of body dissatisfaction. Multiple regression analysis was performed with depression scores and body dissatisfaction as dependent variables. When the depression score was used as the dependent variable, sex, age, muscle mass, BMI, monthly cost of living, exercise intensity score, sleep quality score, and body dissatisfaction level were used as predictor variables. When the level of body dissatisfaction was used as the dependent variable, sex, age, muscle mass, BMI, monthly living expenses, exercise intensity score, and sleep quality score were used as predictor variables. Variables were selected according to the stepwise increase and decrease method, and we used the likelihood ratio test method to calculate and set the threshold *p*-value as 0.20. *p* < 0.05 was considered statistically significant. We used JMP 20.0 J (SAS Institute Inc., Cary, NC) for all statistical analyses and processing.

### 2.9. Ethics approval and consent to participate

Complete research objectives and survey contents were explained to the participants. All participants provided written informed consent, agreeing to the required measurement and survey completion procedures. Ethical approval for the study was granted by the Gannan Medical University, China, No: 2021110.

## 3. Results

**Figure 1:**
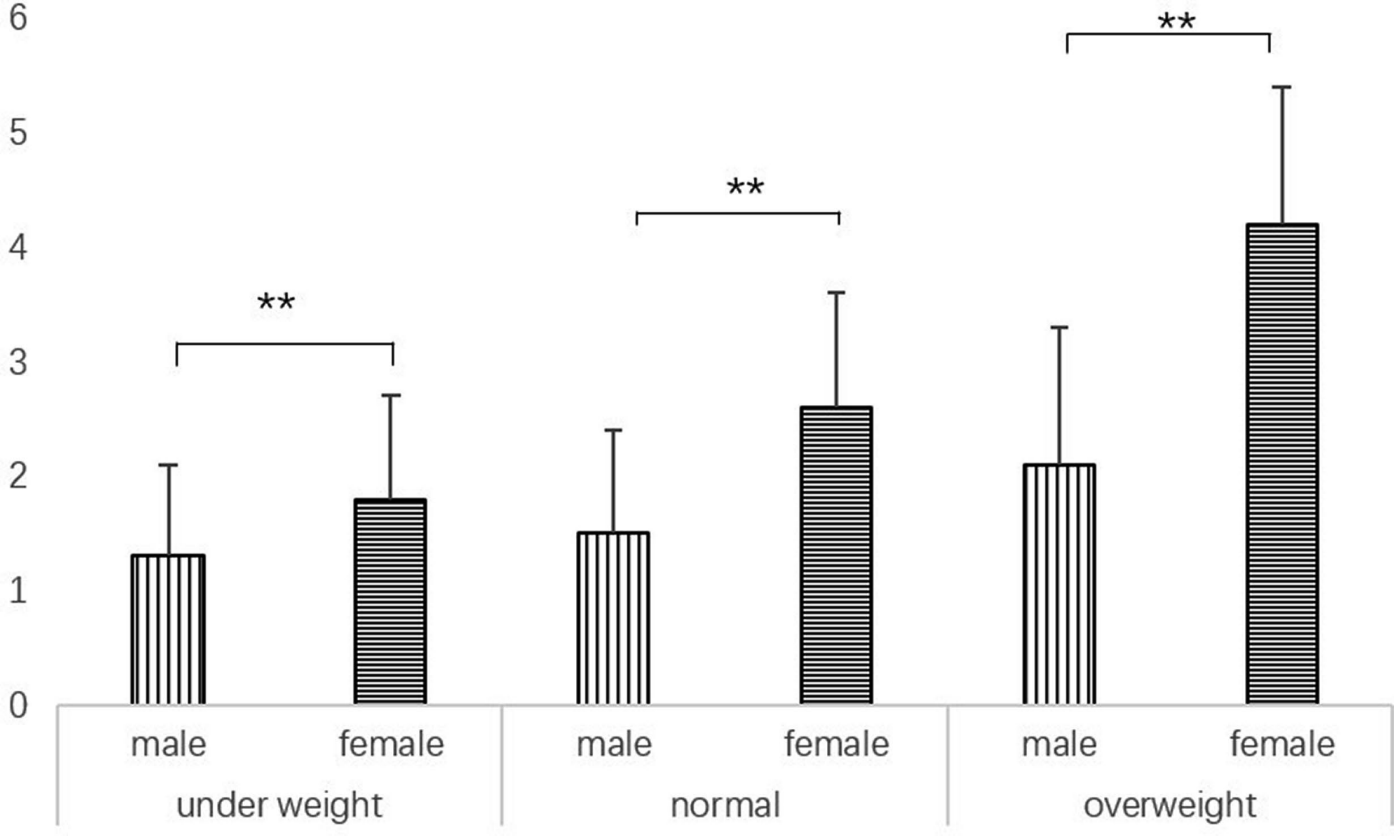
Sex difference of body satisfaction level in college students with different body shapes. ^**^t-test, *p* < 0.01.

Table 1 lists the characteristics of the research subjects. Men had higher BMI and muscle mass than women, while women’s body fat percentage was significantly higher than that of men (*p* < 0.05). There were sex differences in depression, sleep, and exercise scores (Table 1). Women had significantly higher average depression and sleep scores than men (Table 1). Men scored significantly higher than women in terms of physical activity (Table 1). Simultaneously, the level of body dissatisfaction in women in each body type group was significantly higher than that in men (Figure 1).

**Table 1:** Sample characteristics (*n* = 1,258).

	Mean ± SD		*p*
	Male (*n* = 630)	Female (*n* = 628)	
BMI (kg/m^2^)	22.1 ± 3.7	21.2 ± 3.1	<0.01
Fat%	16.1 ± 7.2	27.3 ± 6.6	<0.01
Muscle mass (g)	50.4 ± 8.3	35.9 ± 4.7	<0.01
SDS score	45.2 ± 9.8	50.3 ± 8.9	<0.01
Sleep quality score	16.6 ± 2.5	17.0 ± 2.4	<0.05
Physical activity score	21.5 ± 20.5	12.5 ± 15.4	<0.01
Body dissatisfaction	1.65 ± 1.4	2.71 ± 1.3	<0.01

BMI, body mass index; SDS, Self-rating Depression Scale. The significance of differences between male and female students was determined by *t*-test.

Table 2 shows the results for multiple regression analysis of the factors contributing to the SDS score. Sleep quality score (β = 0.34, *p* < 0.01), sex (β = 0.15, *p* < 0.01), physical activity score (β = −0.14, *p* < 0.01), and body dissatisfaction (β = 0.14, *p* < 0.01) were significant predictive factors of the SDS score.

**Table 2:** Factors that contributed to depression level of university students.

	β	*t*	VIF	*p*
Sleep quality score	0.34	13.48	1.05	<0.01
Sex	0.15	5.59	1.22	<0.01
Physical activity score	−0.14	−5.41	1.11	<0.01
Body dissatisfaction	0.14	4.91	1.29	<0.01

R^2^: 0.25; *p* < 0.01; RMES: 6.74.

The influencing factors of body dissatisfaction obtained through multiple regression analysis are shown in Table 3. BMI (β = 0.30, *p* < 0.01), sex (β = 0.21, *p* < 0.01), sleep quality score (β = 0.19, *p* < 0.01), physical activity score (β = 0.09, *p* < 0.05), and muscle mass (β = 0.09, *p* < 0.05) were significant predictors of body dissatisfaction.

**Table 3:** Factors that contributed to body dissatisfaction of university students.

	β	*t*	VIF	*p*
BMI	0.41	15.45	1.41	<0.01
Sex	0.35	10.08	2.42	<0.01
Sleep quality score	0.16	6.99	1.01	<0.01
Physical activity score	−0.14	−6.17	1.08	<0.01
Muscle mass	−0.08	2.00	2.96	<0.05

R^2^: 0.37; *p* < 0.01; RMES: 1.05.

## 4. Discussion

In recent years, researchers have shown increasing interest in the effects of body image on depression in university students. A study of 160 Hispanic university students showed that the higher the level of body dissatisfaction, the higher was the degree of depression (30). A study in China on 13,046 university students from 17 universities in different regions across the country showed that self-body awareness in early adulthood has been linked to the development of depression (31). The results of our study showed that higher levels of body dissatisfaction were associated with higher levels of depression, supporting the findings of a previous study (Table 2). However, it is worth mentioning that the impact of body dissatisfaction on depression may have been underestimated. The results obtained from the preliminary analysis showed that body dissatisfaction had a less direct effect on depression than exercise and sleep (Table 2). However, an increased level of body dissatisfaction may simultaneously reduce the level of exercise, increase the sleep quality score, and indirectly affect the development of depression (Tables 2, 3).

Prior studies have showed that, among university students, women have higher rates of depression than men. A survey of 286 university students in the United Kingdom revealed a greater risk of depression among women than among men (32). Another survey of 5,989 university students in China showed that the prevalence of depression was higher among women. Our findings are consistent with those of previous studies. The results of the preliminary analysis showed that women had significantly higher depression scores than men (Table 1). The results of this study also show that high body dissatisfaction, low exercise levels, and low sleep quality may lead to depression (Table 2). The low exercise level, sleep quality, and high body dissatisfaction among young women may be important reasons for the high level of depression in women compared to that in men (Tables 1, 2). Body dissatisfaction may also adversely affect exercise level and sleep quality (Table 3). The results of this study highlight that body dissatisfaction may be an important reason for sex differences in depression levels (Tables 2, 3; Figure 1).

Previous studies have shown that regular and moderate physical activity has strong antidepressant effects (33, 34). Additionally, high-intensity exercise improves depression (35). Comparison of the present findings with those of other studies confirms that higher exercise scores among university students in southern China were associated with lower levels of depression (Table 2). Therefore, improving the sports skills of university students is particularly important. For a long time, some researchers tried to develop sports habits and improve participants’ exercise level through exercise interventions, but the effect was limited or unsustainable (36, 37). A note of caution is due here because of the limitation of this simple intervention.

The results of our study indicate that the lower the level of body dissatisfaction among university students, the higher their exercise score (Table 3). Body dissatisfaction is considered an important factor affecting university students’ sports levels. This is consistent with our earlier observations, which showed that if university students are dissatisfied with their body shape, they are less likely to participate in sports (38). University students with a higher degree of body dissatisfaction are more likely to feel embarrassed by outdoor sports (38). They try to reduce outdoor activities as much as possible to avoid being perceived as unattractive (38). Because high exercise levels are a preventative factor for depression (Table 2), improving the level of body satisfaction of university students can increase their exercise level and improve their depression state.

Sleep quality is thought to have an important impact on mental health. A study of 29,099 Chinese university students showed that insomnia is a major risk factor for depression in both sexes (21). According to the results of the preliminary analysis, the lower the sleep quality of university students in southern China, the higher the depression level (Table 2), which supports the results of a previous study. Research has shown that sleep has a lasting negative effect on mental health (39). It is difficult to improve sleep quality with psychotherapy for depression. A study of 465 adolescents in the United Kingdom showed that psychotherapy improved depression but caused limited improvement in sleep quality (6). This may reveal a hidden danger to the recurrence of depression (40). Therefore, further improvement in the management of sleep problems is essential.

We found that the higher the level of body dissatisfaction, the lower the sleep quality of university students (Table 3). Negative or self-critical thinking can reduce sleep quality (6). University students’ steep demands on their bodies and their dissatisfaction with their actual bodies are likely to be important reasons for poor sleep quality. The results of this study showed that body dissatisfaction is common among people of all body types (Figure 1). Therefore, improving body dissatisfaction among university students may have a positive effect on improving the sleep quality of university students. In conclusion, by concatenating the above points, improving university students’ body satisfaction can improve their sleep quality and play a positive role in the prevention and treatment of depression.

### 4.1. Limitations

This study had some limitations. First, the number of participants in this study was small, and their ages ranged from 18 to 23 years. Second, this was a cross-sectional study, and although it showed that body satisfaction may play a positive role in improving depression, it could not clarify the causal relationship. Third, this study only included university students in southern China as the research subjects, and the research results may not be generalizable to university students from other regions. Finally, the survey period overlapped with the period of the COVID-19 pandemic, which may have influenced the study findings.

## 5. Conclusion

The results of this preliminary analysis showed that the depression levels of university students in southern China were significantly higher among females than among males. We found a positive correlation of low body dissatisfaction levels, high physical activity levels, and quality sleep on depression. The findings also emphasize that the role of body dissatisfaction in causing depression may be underestimated. Although improving body satisfaction may directly influence depression levels, it may improve the mood by increasing physical activity and improving sleep quality. Therefore, reducing body dissatisfaction has great potential for preventing and ameliorating depression.

## Data availability statement

The original contributions presented in the study are included in the article/supplementary material, further inquiries can be directed to the corresponding author.

## Ethics statement

The studies involving human participants were reviewed and approved by Gannan Medical University. Written informed consent to participate in this study was provided by the participants’ legal guardian/next of kin.

## Author contributions

MH, XL, YW, QW, WY, and YH involved in the conception of the study and design of the work. YW, WY, and YH involved in the data collection, data analysis, and initial drafting of the manuscript. MH contributed to the interpretation of the analyzed data and critically reviewed the manuscript for important intellectual content. All authors contributed to the article and approved the submitted version.

## Funding

This study was supported by the Starting Research Fund from the Gannan Medical University.

## Conflict of interest

The authors declare that the research was conducted in the absence of any commercial or financial relationships that could be construed as a potential conflict of interest.

## Publisher’s note

All claims expressed in this article are solely those of the authors and do not necessarily represent those of their affiliated organizations, or those of the publisher, the editors and the reviewers. Any product that may be evaluated in this article, or claim that may be made by its manufacturer, is not guaranteed or endorsed by the publisher.
